# Hereditary Tyrosinemia Compounded With Hyperinsulinemic Hypoglycemia: Challenging Diagnosis of a Rare Case

**DOI:** 10.7759/cureus.11541

**Published:** 2020-11-18

**Authors:** Sharmeen Nasir, Mohammad Raza, Samrah I Siddiqui, Ayesha Saleem, Awais Abbas

**Affiliations:** 1 Pediatrics, Dow University of Health Sciences, Karachi, PAK; 2 Pediatrics, Civil Hospital Karachi, Karachi, PAK

**Keywords:** hereditary tyrosinemia, hypoketotic hypoglycemia, hyperinsulinism, metabolic disorder, neonatal hypoglycemia

## Abstract

Hereditary tyrosinemia type 1 (HT-1) is a rare autosomal recessive disorder caused by a deficiency in the enzyme fumarylacetoacetate hydrolase (FAH), which catalyzes the final step in the tyrosine degradation pathway. Hereditary tyrosinemia is a heterogeneous disease with a wide spectrum of clinical manifestations involving hepatic, renal, or nervous systems. It has grave consequences if left untreated. Some of the late complications of hereditary tyrosinemia include cirrhosis, liver nodules, hepatocellular carcinoma, hypophosphatemic rickets, nephrocalcinosis, glomerulosclerosis, and chronic renal failure. Rarely, infants with hereditary tyrosinemia may present with persistent hypoglycemia, which may be a result of acute liver failure or hyperinsulinism. Hyperinsulinemic hypoglycemia (HH), caused by dysregulation of insulin secretion from pancreatic β-cells, leads to insulin driven glucose entry into the tissues and inhibits glycolysis, gluconeogenesis, fatty acid release, and ketone body synthesis. Hyperinsulinemic hypoglycemia can cause severe, persistent hypoketotic hypoglycemia. Diagnosing tyrosinemia type 1 can be a challenge as it is a heterogeneous disorder with a wide variety of clinical manifestations and complications. We herein report a rare case of a three-day-old male neonate with HT-1 compounded with HH.

## Introduction

Hereditary tyrosinemia type 1 (HT-1) is a rare autosomal recessive disorder with an incidence of 1 in 100,000 to 1 in 120,000 live births [1]. HT-1 is caused by a deficiency in the enzyme fumarylacetoacetate hydrolase (FAH) which catalyzes the final step in the tyrosine degradation pathway. This block in the catabolic pathway leads to a buildup of toxic metabolites such as fumarylacetoacetate (FAA), an alkylating agent that causes restricted damage to the liver and kidneys [2,3].

Being a clinically heterogeneous disorder, HT-1 can present at any age from the neonatal period to adulthood with presentation varying even between members of the same family [4,5]. Hepatic involvement usually presents as acute liver failure, coagulopathy, hepatosplenomegaly, ascites, edema, and failure to thrive. The characteristic renal disease is renal tubular acidosis with Fanconi syndrome. Untreated tyrosinemia leads to late complications including cirrhosis, liver nodules, hepatocellular carcinoma, hypophosphatemic rickets, nephrocalcinosis, glomerulosclerosis, and chronic renal failure [4,6].

## Case presentation

A three-day-old male neonate presented to the pediatric ER with a one-day history of lethargy, reluctance to feed, and respiratory distress. He was born of a consanguineous marriage at 39 weeks’ gestation via a normal spontaneous vaginal delivery at home. According to the maternal report, she had no antenatal visits and delivery was uneventful. On further inquiry, it was found that the patient had three male siblings who expired in the early neonatal period, succumbing to similar complaints as that of the patient. The cause of their death was, however, unidentified.

On arrival in the ER, the child was noted to be a plethoric, macrosomic baby with no dysmorphism weighing 4 kilograms (90th percentile) with length and head circumference within the normal limits. On physical examination, the patient looked sick, jaundiced, and drowsy, with the following vital signs: temperature 37°C, respiratory rate 62 breaths per minute, pulse 140 beats per minute, oxygen saturation of 94%, and capillary blood sugar of 22 mg/dL. Palpation of the abdomen revealed a firm, non-tender, enlarged liver, 5 cm below the costal margin. No other viscera were palpable. Neurological examination showed poor neonatal reflexes. Respiratory examination revealed tachypnea, subcostal recessions, and bilateral equal entry of air. The remainder of the physical examination was normal. 

The patient was admitted to the neonatal intensive care unit (NICU) and baseline investigations were run. Complete blood counts and renal function tests returned within the normal ranges. Liver function tests revealed conjugated hyperbilirubinemia (direct bilirubin = 61.5 μmol/L), mildly elevated liver enzymes (alanine aminotransferase (ALT) = 106 U/L), and deranged coagulation profile (international normalised ratio (INR) = 1.8). For detailed results at admission, see Table 1. Results of the laboratory evaluation were notable for hypoglycemia (blood sugar level = 18 mg/dL; 0.999 mmol/L) and a high anion gap metabolic acidosis (anion gap = 24). Blood was drawn during the period of hypoglycemia and sent for insulin levels and serum ketones. First voided urine during the hypoglycemic episode, requesting glucose, ketones, and reducing substances was also sent.

**Table 1:** Initial Laboratory Investigations ALP, alkaline phosphatase; ALT, alanine aminotransferase; APTT, activated partial thromboplastin time; AST, aspartate aminotransferase; GGT, gamma-glutamyltransferase; INR, international normalized ratio; PT, prothrombin time

	Normal	Result
INR	<1.1	1.8
PT (s)	11-14	22
APTT (s)	23-35	32
Direct bilirubin (μmol/L)	<10	61.5
ALP (U/L)	150-420	200
AST (U/L)	47-150	70
ALT (U/L)	13- 45	106
GGT (U/L)	13-147	30
Urea (mmol/L)	0.7-6.7	3.2
Creatinine (μmol/L)	27-88	52
Na+ (mmol/L)	135-145	134
K+ (mmol/L)	3.7-5.9	4
Anion gap (mmol/L)	6-16	24

An intravenous bolus of 10% dextrose at the rate of 2 mL/kg was given to correct the hypoglycemia. Vitamin K was administered to treat the raised INR. Intravenous antibiotics were empirically started to cover for presumed sepsis. Despite the above measures, his hypoglycemic state persisted along with a reluctance to feed, therefore, 10% dextrose at the rate of 60 mL/kg/day was intravenously started. The glucose infusion rate was eventually raised to 17 mg/kg/min to maintain his blood sugar levels. Critical samples sent during the hypoglycemic episode yielded negative serum ketones and a serum insulin level of 5 μU/mL (34.7 pmol/L). Urine samples returned negative for ketones and reducing sugar as well. Results of critical samples are mentioned below in Table 2. Oral diazoxide was started at a dosage of 5 mg/kg/day to treat the hyperinsulinemia. The dosage was later increased to 15 mg/kg/day to meet the patients’ requirements. 

**Table 2:** Critical Samples (Drawn at a Time of Fasting Hypoglycemia: Plasma Glucose <50 mg/dL) "Critical sample" includes the levels of key fuels and hormones required to evaluate for the cause of hypoglycemia.

	Normal	Result
Blood glucose level (mmol/L)	>2.6	0.99
Serum insulin (μU/mL)	<2	5
Serum ketones	Negative	Negative
Urinary ketones	Negative	Negative
Urine for reducing sugar	Negative	Negative
Urine for sugar chromatography	Negative	Negative

Suspicion was high for a metabolic disorder therefore further investigations were run. Urine samples for organic acid chromatography yielded two peaks of succinyl acetoacetate, confirming the diagnosis of hereditary tyrosinemia. Plasma tyrosine and methionine levels were 400 μmol/L and 437 μmol/L, respectively. Serum alpha-fetoprotein (AFP) levels were also raised (AFP = 450 ng/mL). Parents were counseled about the treatment options including dietary management with tyrosine- and phenylalanine-restricted formula and medical management with nitisinone, which is an effective treatment if started early in life. Late complications and the grave consequences, if left untreated, resulting in liver failure and the need for liver transplant in the future were also discussed. At the time of writing the report, the patient was transferred to a tertiary care hospital and was being managed with oral diazoxide at a dosage of 15 mg/kg/day along with a tyrosine- and phenylalanine-restricted formula. The patients' family, however, refused treatment with nitisinone due to the high cost. Genetic counseling was done regarding the risk of disease in future pregnancies and the role of DNA mutation analysis along with measuring succinyl acetoacetate in amniotic fluid during the antenatal period for prenatal diagnosis was highlighted to the family. 

## Discussion

Hereditary tyrosinemia is an inborn error of metabolism with a wide spectrum of clinical manifestations, predominantly targeting the liver, kidneys, and occasionally the nervous system [1]. In a study conducted on 45 patients with type 1 tyrosinemia, Dehghani et al. found that the initial presentation consisted of hepatic involvement in 80% of the patients [4]. The idea that tyrosinemia most commonly affects the liver was also supported by a case report from Pakistan, where two infants presented with hepatomegaly, significantly raised alpha-fetoprotein (AFP), and multiple focal hepatic lesions [7]. Likewise, our patient presented with hepatomegaly, only moderately elevated liver enzymes and raised AFP along with an exceptionally rare manifestation of hyperinsulinemic hypoglycemia (HH). It is noteworthy that infants presenting with liver disease and markedly elevated AFP should be screened for metabolic disorders with hereditary tyrosinemia considered as a differential diagnosis.

Persistent HH has been associated with metabolic disorders such as congenital glycosylation defects and tyrosinemia type 1 [8]. Baumann et al. reported three subjects with tyrosinemia type 1 who had hyperinsulinism in early infancy. The onset of hypoglycemia in these patients was at 84, 63, and 21 days of life, respectively. All three patients reported were successfully treated with diazoxide (10 mg/kg/day) and chlorothiazide (35 mg/kg/day) and the treatment was gradually withdrawn after 9, 13, and 34 months, respectively [9]. In contrast, our patient presented with hypoglycemia on the third day of life and responded to a higher dosage of diazoxide at 15 mg/kg/day. Therefore, it can be presumed that earlier presentations could manifest more severely, requiring more aggressive treatment.

Diagnosing tyrosinemia type 1 can be a challenge as it is a heterogeneous disorder with a wide variety of clinical manifestations and complications. There have been numerous cases reporting a characteristic odor of “boiled cabbage” or “rotten mushrooms” in patients with hereditary tyrosinemia [7,10]. The odor along with elevated AFP, normal or mildly elevated alanine transaminase, which is disproportionate to the level of coagulopathy, can prove to be important clues for reaching the diagnosis [10]. Furthermore, diagnosis cannot be made solely based on elevated tyrosine levels. Marked elevation of succinylacetone (SA) in plasma or urine is considered pathognomonic for the diagnosis of tyrosinemia type 1 [10,11]. DNA mutation analysis of the FAH gene and enzymatic analysis of FAH in cultured skin fibroblasts confirms the diagnosis of tyrosinemia type 1 [10]. Confirmatory testing with these modalities is not readily available and seldom required as the diagnosis can be confidently established by elevated succinyl acetone levels. Measuring SA during the antenatal period in amniotic fluid or as part of the neonatal screening program in combination with the screening of at-risk siblings may lead to an improved prognosis due to early diagnosis and initiation of treatment [2,11]. 

Survival of patients with HT-1 has radically improved with the use of nitisinone, 2-(2-nitro-4-trifluoromethylbenzoyl)-1,3-cyclohexanedione (NTBC), a pharmacological agent that inhibits the formation of toxic tyrosine intermediates by reversible inhibition of 4-hydroxypheylpyruvate dioxygenase (HPPD) [6]. NTBC is recommended to be used in combination with a norm caloric tyrosine- and phenylalanine-restricted diet. This treatment controls liver disease in 90% of the patients and resolves non-hepatic manifestations. It has also been particularly successful in preventing the risk of hepatic malignancies [11,12]. NTBC, now being the first-line treatment, is indicated in all patients with HT-1. Liver transplantation is indicated when either treatment with NTBC fails or the development of hepatocellular carcinoma is likely [11]. However, the high cost of this treatment limits its use in a resource-poor country like ours, posing a challenge to its management.

## Conclusions

This report describes an association of two rare disorders, hereditary tyrosinemia type 1 and hyperinsulinemic hypoglycemia. Diagnosing tyrosinemia type 1 poses a great challenge due to its heterogeneous manifestations. We suggest keeping a high index of suspicion for metabolic disorders in young infants with hepatomegaly, unexplained metabolic acidosis, severe persistent hypoglycemia, and a family history of multiple expiries due to unknown cause to prevent long-term complications and early mortality. Elevated levels of succinyl acetoacetate in plasma or urine via gas chromatography/mass spectroscopy confirm the diagnosis of hereditary tyrosinemia type 1. Clinicians may have to specially request for succinylacetone so that selective ion monitoring is incorporated into the laboratory analysis for its detection. NTBC combined with a tyrosine- and phenylalanine-restricted diet is considered the cornerstone of the medical management in HT-1. Liver transplant has opted when treatment with NTBC fails or there is a high index for the development of hepatocellular carcinoma. These expensive treatment options warrant incorporating practices of measuring succinyl acetoacetate levels during the antenatal period, in couples at risk of having children with HT-1, especially in a resource-poor country like Pakistan.
